# The RENAISSANCE (AIO-FLOT5) trial: effect of chemotherapy alone vs. chemotherapy followed by surgical resection on survival and quality of life in patients with limited-metastatic adenocarcinoma of the stomach or esophagogastric junction – a phase III trial of the German AIO/CAO-V/CAOGI

**DOI:** 10.1186/s12885-017-3918-9

**Published:** 2017-12-28

**Authors:** Salah-Eddin Al-Batran, Thorsten O. Goetze, Daniel W. Mueller, Arndt Vogel, Michael Winkler, Sylvie Lorenzen, Alexander Novotny, Claudia Pauligk, Nils Homann, Thomas Jungbluth, Christoph Reissfelder, Karel Caca, Steffen Retter, Eva Horndasch, Julia Gumpp, Claus Bolling, Karl-Hermann Fuchs, Wolfgang Blau, Winfried Padberg, Michael Pohl, Andreas Wunsch, Patrick Michl, Frank Mannes, Matthias Schwarzbach, Harald Schmalenberg, Michael Hohaus, Christian Scholz, Christoph Benckert, Jorge Riera Knorrenschild, Veit Kanngießer, Thomas Zander, Hakan Alakus, Ralf-Dieter Hofheinz, Claus Roedel, Manish A. Shah, Mitsuru Sasako, Dietmar Lorenz, Jakob Izbicki, Wolf O. Bechstein, Hauke Lang, Stefan P. Moenig

**Affiliations:** 1 Institute of Clinical Cancer Research (IKF) at Krankenhaus Nordwest, UCT-University Cancer Center, Krankenhaus Nordwest, Steinbacher Hohl 2-26, 60488 Frankfurt am Main, Germany; 2 Department of Internal Medicine, Hannover Medical School, 30625 Hannover, Germany; 3 Department of Surgery, Hannover Medical School, 30625 Hannover, Germany; 4 Department of Internal Medicine, Klinikum rechts der Isar der TU München, 81675 Munich, Germany; 5 Department of Surgery, Klinikum rechts der Isar der TU München, 81675 Munich, Germany; 6 Department of Internal Medicine II, Academic Teaching Hospital Wolfsburg, 05361 Wolfsburg, Germany; 7 Department of Surgery, Academic Teaching Hospital Wolfsburg, 05361 Wolfsburg, Germany; 8 Department of Surgery, University Hospital Carl Gustav Carus Dresden, 01307 Dresden, Germany; 9 Department of Internal Medicine, Klinikum Ludwigsburg, 71640 Ludwigsburg, Germany; 10 Department of Surgery, Klinikum Ludwigsburg, 71640 Ludwigsburg, Germany; 11 Department of Internal Medicine, Kliniken des Landkreises Neumarkt, 92318 Neumarkt, Germany; 12 Department of Surgery, Kliniken des Landkreises Neumarkt, 92318 Neumarkt, Germany; 13 Department of Internal Medicine, Agaplesion Markus Krankenhaus Frankfurter, Diakonie Kliniken gGmbH, 60431 Frankfurt, Germany; 14 Department of Surgery, Agaplesion Markus Krankenhaus Frankfurter Diakonie Kliniken gGmbH, 60431 Frankfurt, Germany; 15 Department of Medical Oncology, Gießen University Hospital, 35392 Gießen, Germany; 16 Department of Surgery, Gießen University Hospital, 35392 Gießen, Germany; 17 Department of Internal Medicine, Ruhr-University Bochum, 44801 Bochum, Germany; 18 Department of Surgery, Ruhr-University Bochum, 44801 Bochum, Germany; 19 Department of Medical Oncology, Halle University Hospital, 06120 Halle (Saale), Germany; 20 Department of Internal Medicine, Halle University Hospital, (Saale), 06120 Halle, Germany; 21 Department of Surgery, Klinikum Frankfurt Höchst, 65929 Frankfurt, Germany; 22 Department of Internal Medicine IV, Städtisches Klinikum Dresden, 01067 Dresden, Germany; 23 Department of Surgery, Städtisches Klinikum Dresden, 01067 Dresden, Germany; 24 Department of Medical Oncology, Vivantes Klinikum Am Urban Berlin, 10967 Berlin, Germany; 25 Department of Surgery, Vivantes Klinikum Am Urban Berlin, 10967 Berlin, Germany; 26 Department of Medical Oncology, Marburg University Hospital, 35043 Marburg, Germany; 27 Department of Surgery, Marburg University Hospital, 35043 Marburg, Germany; 28 Department of Internal Medicine, University Hospital Köln, 50937 Köln, Germany; 29 Department of Surgery, University Hospital Köln, 50937 Köln, Germany; 30 University Medical Center Mannheim, 68167 Mannheim, Germany; 31 Department of Radiation- Oncology, Frankfurt University Hospital, 60590 Frankfurt, Germany; 32 Department of Medicine Hematology and Oncology, Weill Cornell Medicine, New York, USA; 33 Department of Surgery, Hyogo College of Medicine, Mukogawa-cho, Nishinomiya, Hyogo Japan; 34 Department of General and Visceral Surgery, Sana- Klinikum Offenbach, 63069 Offenbach, Hamburg, Germany; 35 Department of Surgery, Hamburg University Hospital, 20246 Hamburg, Germany; 36 Department of Surgery, Frankfurt University Hospital, 60590 Frankfurt, Hamburg, Germany; 37 Department of Surgery, Mainz University Hospital, 55131 Mainz, Germany; 38 Hôpitaux Universitaires de Genève, Service de Chirurgie viscéral, 1205 Genève, Switzerland

**Keywords:** Oligometastatic cancer, Metastatic gastric cancer, Metastatic gastroesophageal junction cancer, Limited-metastatic disease, Localized peritoneal carcinomatosis, Perioperative chemotherapy, FLOT- regimen, Gastrectomy, Resection of metastases, Quality of life

## Abstract

**Background:**

Historical data indicate that surgical resection may benefit select patients with metastatic gastric and gastroesophageal junction cancer. However, randomized clinical trials are lacking. The current RENAISSANCE trial addresses the potential benefits of surgical intervention in gastric and gastroesophageal junction cancer with limited metastases.

**Methods:**

This is a prospective, multicenter, randomized, investigator-initiated phase III trial. Previously untreated patients with limited metastatic stage (retroperitoneal lymph node metastases only or a maximum of one incurable organ site that is potentially resectable or locally controllable with or without retroperitoneal lymph nodes) receive 4 cycles of FLOT chemotherapy alone or with trastuzumab if Her2+. Patients without disease progression after 4 cycles are randomized 1:1 to receive additional chemotherapy cycles or surgical resection of primary and metastases followed by subsequent chemotherapy. 271 patients are to be allocated to the trial, of which at least 176 patients will proceed to randomization. The primary endpoint is overall survival; main secondary endpoints are quality of life assessed by EORTC-QLQ-C30 questionnaire, progression free survival and surgical morbidity and mortality. Recruitment has already started; currently (Feb 2017) 22 patients have been enrolled.

**Discussion:**

If the RENAISSANCE concept proves to be effective, this could potentially lead to a new standard of therapy. On the contrary, if the outcome is negative, patients with gastric or GEJ cancer and metastases will no longer be considered candidates for surgical intervention.

**Trial registration:**

The article reports of a health care intervention on human participants and is registered on October 12, 2015 under ClinicalTrials.gov Identifier: NCT02578368; EudraCT: 2014–002665-30.

## Background

In metastatic stages of gastric cancer, surgical intervention with curative or life-prolonging intention has been evaluated in several subgroup analyses of clinical trials and retrospective patient cohorts. The data obtained indicated, that surgical resection could provide a benefit for selected patient groups such as patients aged 70 years or less with one metastatic site only [1], patients with one metastatic site (lymph nodes or liver) and excellent response to systemic preoperative chemotherapy [2, 3], or patients with metastases limited to the liver, in whom complete resection seems feasible after careful preoperative staging [4]. A German group analyzed 48 patients who underwent primary stomach resection and identified D3 as an independent (positive) predictor of survival [5]. A Japanese group evaluated 16 patients with pathologically positive para-aortal lymph node involvement who underwent curative surgical resection after two cycles of pre-operative docetaxel, cisplatin and S1 chemotherapy. 2-years overall and relapse-free survival rates were 93.8% and 75.0%, respectively [6]. Similar results were reported in multiple case reports [7–9]. Nevertheless, surgical resection remained highly debatable, since randomized trials have been lacking.

A pilot study of our group [10] was able to establish a clinical model to identify a patient population, which could potentially benefits from surgical intervention after induction chemotherapy. Patients with untreated gastric or junctional cancers were prospectively stratified into 3 groups: operable (M0) patients, limited metastatic, or extensive metastatic patients, using a predefined algorithm and treated with FLOT (5-flourouracil, leucovorin, oxaliplatin, and docetaxel). Limited metastatic disease was defined as: distant intra-abdominal lymph node metastases only or/and a maximum of 1 organ involved, normal serum alkaline phosphatase, < 5 liver lesions, no visible carcinomatosis (peritoneum or pleura), and ECOG ≤1. All other metastatic patients were considered extensive. Patients with M0 disease received 4 preoperative FLOT cycles followed by surgery and 4 postoperative cycles. Patients with limited metastatic disease received 4 cycles followed by resection of the primary and metastases if possible. Four additional postoperative cycles were administered. Patients with extensive metastatic disease received 8 cycles with surgery allowed for palliation only. 60 out of 238 patients enrolled had limited metastatic stage. Thirty-six of them (60%) proceeded to surgery after FLOT chemotherapy. The study observed a considerable median overall survival of 31 months for the resected patients with limited metastatic stage (versus 16 months for patients without resection) and provided the rational for the present study.

## Methods/design

### Protocol overview

**Fig. 1:**
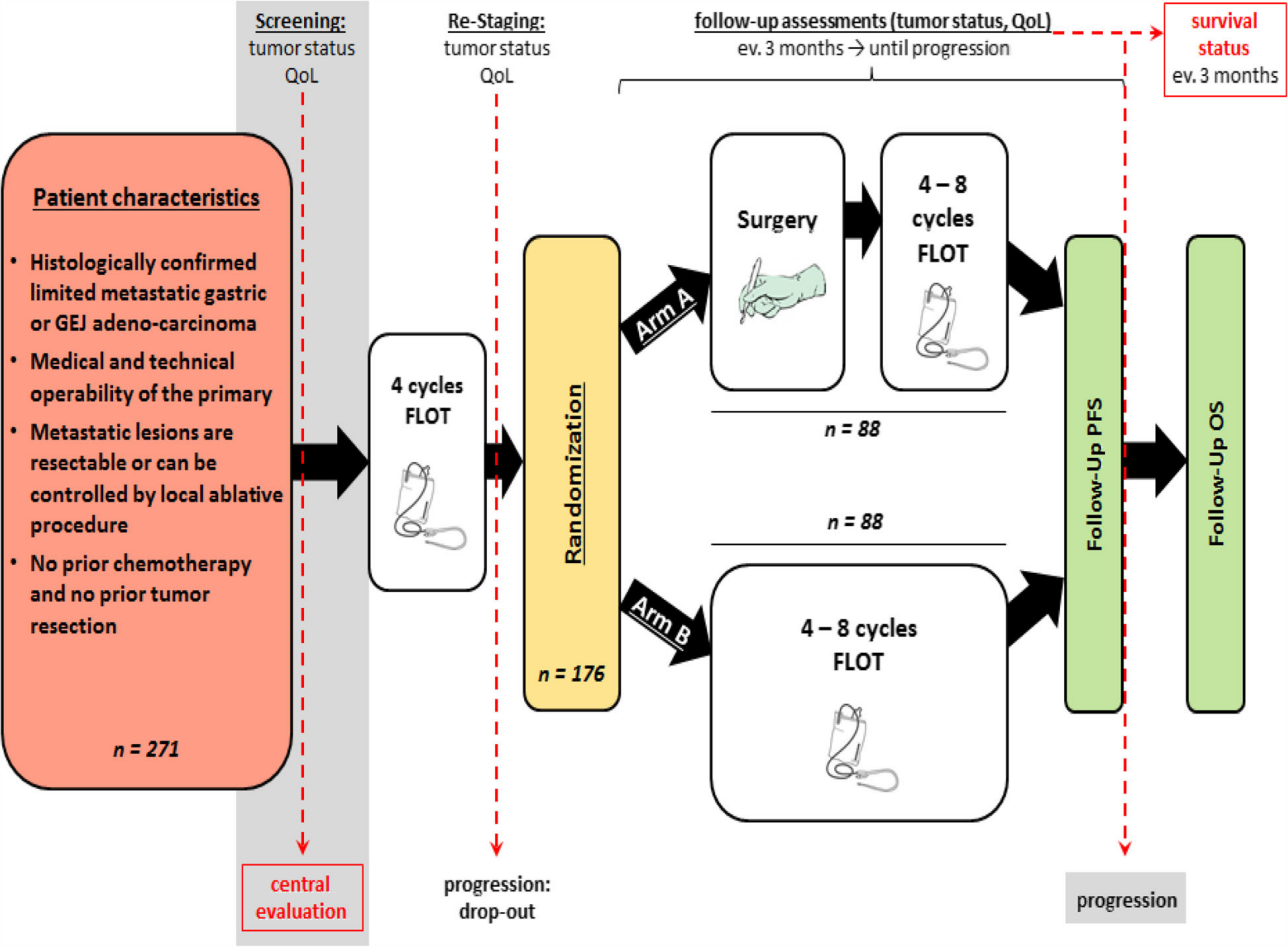
RENAISSANCE study flow chart (schematic)

RENAISSANCE is a prospective, multicenter, randomized, investigator-initiated phase III trial aimed to evaluate the effects of perioperative chemotherapy with FLOT in chemo naïve patients with limited metastatic (exact definition see next section) gastric/GEJ cancer (without prior tumor resection) in combination with curative gastrectomy/esophagectomy + resection of metastatic lesions or local ablation procedure (Fig. 1 – Study flow chart). Patients with potentially limited metastatic gastric cancer or adenocarcinoma of the GEJ potentially fulfilling the selection criteria (detailed inclusion and exclusion criteria see section below) and who gave informed consent will undergo a careful screening and a central review process (details see below). Patients who fulfill all eligibility criteria and are positively evaluated in a central review will be enrolled into the study. All patients enrolled will receive four cycles (= 8 weeks) of FLOT [Docetaxel 50 mg/m^2^, iv over 2 h, d1; Oxaliplatin 85 mg/m^2^ in 500 ml G5%, iv over 2 h, d1; Leucovorin 200 mg/m^2^ in 250 ml NaCl 0.9%, iv over 1 h, d1; 5-FU 2600 mg/m^2^, iv over 24 h, d1 (= 1 cycle); Start of next cycle on day 15 (every two weeks)] [11] . For HER-2 positive disease, trastuzumab will be added. Treatment will be administered on day one of biweekly cycles. After the 4th cycle of FLOT, patients will undergo a repeated imaging (esophago-gastro-duodenoscopy, CT/MRI or PET scan of the involved organs). Patients with disease progression will be taken out of the trial. Patients with stable disease, partial or complete remission will be stratified by tumor location (gastric vs. GEJ adenocarcinoma), response to preoperative FLOT (complete or partial remission vs. stable disease) and based on whether they have distant lymph node metastases only or additional organ involvement and will be randomized 1:1 to Arm A (with surgery) or B (no surgery).

#### Arm a:

Surgery will be scheduled 4–6 weeks after d1 of the last cycle of preoperative chemotherapy (d1 + 4–6 weeks). The type of surgical procedure is determined by the location and extent of the primary tumor and should be performed according to local standards. In terms of the metastatic disease, the protocol provides recommendations on surgical intervention for the different types of limited metastatic disease (type 1 to 2.VII). Post-operatively, further 4–8 cycles of FLOT can be administered starting 4 to 12 weeks after surgery. If additional local ablation procedures are considered, these can be performed in parallel with the postoperative chemotherapy if this is considered feasible by the investigator.

#### Arm B:

Patients will be treated with additional 4–8 cycles of FLOT. Surgical interventions are allowed for palliation. In both arms, the continuation of chemotherapy for more than 12 cycles of FLOT or de-escalated/modified FLOT such as FLO or FLT is possible, if the investigator believes that this in the best interest of the patient. A maintenance therapy using one or more of the FLOT components (oral forms also permitted) is permitted and can be performed according to local guidelines.

In both of the arms, tumor assessments (CT/MRI or PET of the relevant organs) are performed prior to randomization and then every 3 months thereafter until progression/relapse, death or end of follow-up. During chemotherapy, clinical visits (blood cell counts, detection of toxicity) occur every two weeks, in general prior to the chemotherapy administration.

Quality of life (QoL) will be assessed using the European Organization for Research and Treatment of Cancer Quality of Life Questionnaire-C30 (EORTC-QLQ-C30) at baseline, prior to randomization, and every 3 months after randomization during treatment and in the follow-up phase, together with tumor assessments. QoL assessment also ends with disease progression/relapse, death or end of follow-up. After disease progression/relapse, survival status will be assessed every 3 months for up to 5 years after randomization.

#### Definition of limited metastatic status according Flot3-study with modification:

Retroperitoneal lymph node metastases (RPLM) only (e.g., para-aortal, intra-aorto-caval, parapancreatic or mesenterial lymph nodes);

- Note: in duodenum invading gastric cancer, retropancreatic nodes are not regarded M1 or/and at maximum one organ involved with or without RPLM according to the following schema:
- Localized potentially operable peritoneal carcinomatosis: stage P1 according to classification of the “Japanese Research Society for Gastric Cancer” (Clinically visible carcinomatosis of the peritoneum or of the pleura and >P1 peritoneal carcinomatosis are not allowed!) or
- Liver: maximum of 5 metastatic lesions that are potentially resectable or
- Lung: unilateral involvement, potentially resectable or
- Uni- or bilateral Krukenberg tumors (ovarian met.) in the absence of macroscopic peritoneal carcinomatosis or
- Uni- or bilateral adrenal gland metastases or
- Extra-abdominal lymph node metastases such as supraclavicular or cervical lymph node involvement or
- Localized bone involvement (defined as being within one radiation field) or
- Other metastatic disease location that is considered limited by the investigator and is confirmed by the review committee

### Measures of outcomes and assessments

#### Primary outcome

Overall survival is the primary endpoint. The duration of OS will be determined by measuring the time interval from randomization to the date of death or last observation (censored).

#### Secondary outcomes

QoL is the main secondary endpoint. The QoL data will help us to better integrate a potential gain in OS into the treatment guidelines. Other secondary outcome measures are 1-, 2- and 3-year survival rates as well as the projected 5-year overall survival rate in addition to progression-free survival, toxicity, 30 days and 90 days (perioperative) morbidity and mortality.

#### Main inclusion criteria

Histologically confirmed limited metastatic (definition) gastric or GEJ adenocarcinoma. Medical and technical operability of the primary (central evaluation). Metastatic lesions are resectable or can be controlled by local ablative procedure (central evaluation). No prior chemotherapy and no prior tumor resection.

#### Main exclusion criteria

Medical inoperability. Inability to understand the study and/or comply with the protocol procedures. Extensive metastatic status or cM0. Secondary malignancy <3 years ago.

### Treatments

#### Control(s)/comparator(s)

FLOT consists of: Docetaxel 50 mg/m^2^, iv over 2 h, d1; Oxaliplatin 85 mg/m^2^ in 500 ml G5%, iv over 2 h, d1; Leucovorin 200 mg/m^2^ in 250 ml NaCl 0.9%, iv over 1 h, d1; 5-FU 2600 mg/m^2^, iv over 24 h, d1 (= 1 cycle); Start of next cycle on day 15 (every two weeks). [11]

#### Dose, mode and scheme of intervention

In the interventional arm, patients will undergo surgery 4 to 6 weeks after the 4th cycle of FLOT, as done and found safe in the Flot3 and Flot4 trials [10, 11]. Additional 4 to 8 cycles of FLOT are to be administered, starting 4 to 12 weeks after surgery. Goal of surgery is a complete (R0 and at least D2) resection of the primary tumor including standardized lymphadenectomy and, whenever technically possible, complete (R0) resection or complete macroscopic cytoreduction of the metastases.

#### Sample size calculation

The primary efficacy analysis will compare randomized chemotherapy-alone to randomized chemotherapy followed by surgical resection on the time to the primary efficacy endpoint using the ITT population. The hypothesis test will use the log rank test to compare the investigational arms. The study assumes a Hazard ratio of 0.65 favoring the surgery group. The OS in the reference arm is set as 16 months. Accrual time is 4 years followed by a 2 years follow up period. Dropouts prior to randomization are set at 35%. Dropouts after randomization are set 10%. Type I error is 5% and one-sided Log rank test is used. 271 patients will be enrolled and 176 patients are to be randomized to provide a statistical power of 80%.

### Ethical considerations, information giving and written informed consent

The study protocol was approved by the responsible lead ethics committee on the 11th of January 2016 under the identification number FF123/2015. The study has been registered on the ClinicalTrial.gov website under the identification number NCT02578368. The RENAISSANCE study complies with the Declaration of Helsinki rules, the principles of Good Clinical Practice guidelines and the Data Protection Act. The trial will also be carried out in compliance to local legal and regulatory requirements. For each patient to be enrolled into the study, obtaining written informed consent prior to inclusion into the study is essential.

## Discussion

Recent data indicates that surgical resection [1] may have benefit for selected patients with metastatic gastric or gastroesophageal junction cancer [2, 3], but randomized trials are still lacking. Therefore, surgical resection remains highly debatable up to date. The current RENAISSANCE trial investigates the question about the role of surgical intervention in limited-metastatic gastric and GEJ cancer. If the concept proves to be effective, this could potentially lead to a new standard of care with direct benefits to cancer patients. On the contrary, if the outcome of the study is negative, patients with metastatic gastric or GEJ cancer should no longer be considered candidates for surgical intervention as they are currently in some constellations. This will help to preserve the QoL of these patients as well as to lower the morbidity associated with an ineffective surgery and will be accompanied by cost savings for the public and private health insurance systems.

A recent randomized, Asian trial (*n* = 175) enrolled patients with gastric cancer who had a single non-curable site (liver, peritoneum, or para-aortic lymph nodes) to chemotherapy alone or gastrectomy followed by chemotherapy [12]. The study failed to show improvements in survival by surgery. In the contrary, results showed a trend towards inferiority in the surgery group (median OS was 16.6 months in patients without versus and 14.3 months in patients with gastrectomy).

In contrast to the trial mentioned above, our study is based on three theoretical aspects that we consider very important in the context of implementing surgical resection for metastatic patients: first, the proper selection of candidates who are more likely to benefit from local therapy, such as patients with favorable prognostic factors (e.g. performance status) and factors related to the type and extent of metastatic involvement. In the future, biology also has to be considered; second, the clear definition of the goal of surgery, which is curative and not palliative in our setting; and third, the necessity to administer effective systemic chemotherapy prior to and after surgery. The administration of upfront chemotherapy is important because gastric cancer is a biologically aggressive disease. The lack of upfront chemotherapy would cause a delay in administration of the effective systemic treatment component in the surgery group, thus negatively affecting survival in the surgery population.

The use of FLOT chemotherapy is supported by numerous reports indicating that FLOT is superior to other regimen such as FOLFOX (5-FU, leucovorin, and oxaliplatin) or ECF (epirubicin, cisplatin, and 5-FU) in terms of pathological regression, which is regarded important in the context addressed in our trial. [11, 13, 14]

The sample size calculation is based on a hazard ratio of 0.65 that seems very ambitious. However, the implementation of a major surgery (gastrectomy and/or esophagectomy) is a very burdensome intervention with high impact on patient’s quality of life and physical function that is barely justified by small improvements of survival, usually perused in drug research.

Finally, there are significant challenges facing our trial. The target population is relatively small. Some investigators and their surgeons have great difficulties randomizing patients with very limited stage such as retroperitoneal lymph nodes or single liver metastases to a non-surgery arm. This will not only slow recruitment, it will also inflate the study by high risk patients. We also expect that many patients will refuse participation or will cross-over after randomization because they want to participate at decision making. We implemented several processes to cope with these challenges, including but are not limited to a very high dropout rate of 45% (35% prior to and 10% after randomization) and a comprehensive communication plan with centers to ensure that the study is explained to the patients in a fair and appropriate way. We also planned a long recruitment period of four years.

## Abbreviations

AIO: Arbeitsgemeinschaft Internistische Onkologie- Working group of Medical Oncologists
DFG: Deutsche Forschungsgemeinschaft (German Research Foundation)
EORTC: European Organization for Research and Treatment of Cancer
EORTC-QLQ-C30: European Organization for Research and Treatment of Cancer Quality of Life Questionnaire-C30
FLOT: 5-flourouracil, leucovorin, oxaliplatin, and docetaxel
GEJ: gastroesophageal junction cancer
QoL: Quality of Life

## References

[1] Hartgrink HH (2002). Value of palliative resection in gastric cancer. Br J Surg.

[2] Yoshida M (2004). Long-term survival and prognostic factors in patients with metastatic gastric cancers treated with chemotherapy in the Japan clinical oncology group (JCOG) study. Jpn J Clin Oncol.

[3] Lee JH (2006). Candidates for curative resection in advanced gastric cancer patients who had equivocal para-aortic lymph node metastasis on computed tomographic scan. Ann Surg Oncol.

[4] Cheon SH (2008). Survival benefit of combined curative resection of the stomach (D2 resection) and liver in gastric cancer patients with liver metastases. Ann Onc.

[5] Dittmar Y (2012). Non-curative gastric resection for patients with stage 4 gastric cancer--a single center experience and current review of literature. Langenbeck's Arch Surg.

[6] Oyama K (2012). Efficacy of pre-operative chemotherapy with docetaxel, cisplatin, and S-1 (DCS therapy) and curative resection for gastric cancer with pathologically positive para-aortic lymph nodes. J Surg Oncol.

[7] Kanda T (2012). Gastrectomy as a secondary surgery for stage IV gastric cancer patients who underwent S-1-based chemotherapy: a multi-institute retrospective study. Gastric Cancer.

[8] Tanaka C (2010). Three cases of gastric cancer with para-aortic lymph node metastases succesfully treated by S-1/CDDP combination therapy followed by curative resection. Gan To Kagaku Ryoho.

[9] Suzuki Y (2009). A case of marked response to CPT-11+CDDP neoadjuvant chemotherapy for advanced gastric cancer with paraaortic lymph node metastasis enabling curative resection and over 10-year survival. Gan To Kagaku Ryoho..

[10] Al-Batran SE, et al. Neoadjuvant chemotherapy followed by surgical resection for patients with limited metastatic gastric or gastroesophageal junction cancer: results from the AIO-FLOT3 trial. JAMA Oncology. in press; 10.1001/jamaoncol.2017.0515 PMC5824287 28448662

[11] Al-Batran SE (2016). Histopathological regression after neoadjuvant docetaxel, oxaliplatin, fluorouracil, and leucovorin versus epirubicin, cisplatin, and fluorouracil or capecitabine in patients with resectable gastric or gastro-oesophageal junction adenocarcinoma (FLOT4-AIO): results from the phase 2 part of a multicentre, open-label, randomised phase 2/3 trial. Lancet Oncol.

[12] Fujitani K (2016). Gastrectomy plus chemotherapy versus chemotherapy alone for advanced gastric cancer with a single non-curable factor (REGATTA): a phase 3, randomised controlled trial. Lancet Oncol..

[13] Homann N (2012). Pathological complete remission in patients with oesophagogastric cancer receiving preoperative 5-fluorouracil, oxaliplatin and docetaxel. Int J Cancer.

[14] Schulz C (2015). NeoFLOT: multicenter phase II study of perioperative chemotherapy in resectable adenocarcinoma of the gastroesophageal junction or gastric adenocarcinoma—very good response predominantly in patients with intestinal type tumors. Int J Cancer.

